# Case Report: Necrotizing fasciitis caused by *Staphylococcus aureus* positive for a new sequence variant of exfoliative toxin E

**DOI:** 10.3389/fgene.2022.964358

**Published:** 2022-09-15

**Authors:** Artur J. Sabat, Marjan Wouthuyzen-Bakker, Angelique Rondags, Laura Hughes, Viktoria Akkerboom, Olga Koutsopetra, Alexander W. Friedrich, Erik Bathoorn

**Affiliations:** ^1^ Department of Medical Microbiology and Infection Prevention, University Medical Center Groningen, University of Groningen, Groningen, Netherlands; ^2^ Department of Dermatology, University Medical Center Groningen, University of Groningen, Groningen, Netherlands

**Keywords:** necrotizing fasciitis, *Staphylococcus aureus*, ETE, ETE2, PVL

## Abstract

**Objectives:** Necrotizing fasciitis (NF) caused by *S. aureus* is a rare, aggressive and rapidly progressing superficial fascia infection with a high mortality rate. The aim of this study was to identify virulence-related genes from a complete genome sequence of a methicillin-susceptible *S. aureus* (MSSA) isolate recovered from a monomicrobial case of NF.

**Materials and methods:** The MSSA isolate UMCG579 was cultured from a pus collection from the subcutis of a patient with NF. The genome of isolate UMCG579 was sequenced using MinION (Oxford Nanopore) and MiSeq (illumina) platforms.

**Results:** The genome of the UMCG579 isolate was composed of a 2,741,379 bp chromosome and did not harbor any plasmids. Virulence factor profiling identified multiple pore-forming toxin genes in the UMCG579 chromosome, including the Panton-Valentine leukocidin (PVL) genes, and none of the superantigen genes. The UMCG579 isolate harbored a new sequence variant of the recently described *ete* gene encoding exfoliative toxin (type E). A search in the GenBank database revealed that the new sequence variant (*ete2*) was exclusively found among isolates (*n* = 115) belonging to MLST CC152. While the majority of *S. aureus ete*-positive isolates were recovered from animal sources, *S. aureus ete2*-positive isolates originated from human carriers and human infections. Comparative genome analysis revealed that the *ete2* gene was located on a 8777 bp genomic island.

**Conclusion:** The combination of two heterogeneously distributed potent toxins, ETE2 and PVL, is likely to enhance the pathogenic ability of *S. aureus* isolates. Since anti-virulence therapies for the treatment of *S. aureus* infections continue to be explored, the understanding of specific pathogenetic mechanisms may have an important prophylactic and therapeutic value. Nevertheless, the exact contribution of ETE sequence variants to *S. aureus* virulence in NF infections must be determined.

## Introduction

Necrotizing fasciitis (NF) is a rare but severe infection characterised by necrosis of the superficial fascia with a high mortality rate (25%–30% cases). It is a surgical diagnosis. NF can rapidly progress to life-threatening septic shock. Symptoms can be non-specific at first, but classically present with swelling and erythema (Goh et al., 2014). Moreover, intense pain disproportionate to the results of the physical examination may be observed. The progression of NF can be characterised by the development of clear bullae, crepitus and skin necrosis (Kiat et al., 2017). The presence of skin lesions (bullae) has been shown to have a positive relationship with the rate of amputation and mortality, suggesting that this symptom may be regarded as a turning point in the prognosis of the disease (Karnuta et al., 2020). As the infection progresses, toxins are released spreading into the bloodstream. It can lead to severe systemic toxicity, which can eventually result in multi-organ failure. There are a number of established risk factors for disease including an immunocompromised status, diabetes, smoking, alcohol abuse, and the use of injectable drugs. However, disease can also develop in people of all ages without underlying conditions (Burnham et al., 2016; Stevens et al., 2021). Once NF is clinically highly suspected prompt surgical intervention is needed for confirmation of diagnosis and for treatment in combination with appropriate antimicrobial treatment to reduce mortality and improve outcomes. Patients who survive often suffer from severe complications, the most common of which include; nosocomial infections, respiratory distress syndrome, acute renal failure and limb amputation.

There are two main types of NF (Stevens and Bryant, 2017). Type I is characterised by polymicrobial infections and accounts for approximately 75% of all cases. Type I NF most commonly affects elderly patients and patients with underlying medical conditions. It can be caused by a mixture of aerobic bacteria (such as *Streptococcus* or *Enterobacteriaceae*) and anaerobic bacteria (*Bacteroides* or *Peptostreptococcus*). Type II NF accounts for approximately 25% of all cases and can affect all age groups and also individuals without underlying disease. It is characteristically monomicrobial by nature. *Streptococcus pyogenes* is the major etiological agent of type II NF, sometimes in combination with *Staphylococcus aureus*. *S. aureus* is an uncommon causative agent of monomicrobial NF, but the number of cases caused by this pathogen is increasing (Kao et al., 2011; Thapaliya et al., 2015; Kim et al., 2019). Some experts have proposed type III NF, in which *Vibrio vulnificus*, *Aeromonas hydrophila* and possibly clostridial species are the etiological agents (Stevens and Bryant, 2017).

In NF there is a strong association between exotoxin production at the site of infection and extensive tissue necrosis and systemic toxicity (Shumba et al., 2019). *S. aureus* possesses an extensive arsenal of exotoxins, which can be divided into three major groups: pore-forming toxins (PFTs), exfoliative toxins (ETs), and superantigens (SAgs) (Oliveira et al., 2018). PFTs are the most common bacterial cytotoxic proteins. They disrupt host cell membranes causing cell lysis but also have additional immuno-modulatory effects. ETs are highly specific serine proteases that cleave desmoglein 1 (Amagai et al., 2000; Amagai et al., 2002), a major transmembrane component of desmosomes. This unique proteolytic attack results in a dissociation of keratinocytes in skin, which allows the bacteria to spread beneath this barrier. To date, five types of ETs have been distinguished (ETA, ETB, ETC, ETD, and ETE) with sequence identity ranging from 43% to 63% (Ullah et al., 2022). ETA, ETB, ETD, and ETE are implicated in human skin damage, while ETC is not toxic to humans and has only been recovered from a horse infection (Oliveira et al., 2018; Imanishi et al., 2019; Azarian et al., 2021; Ullah et al., 2022). SAgs constitute exotoxins that stimulate extensive T cell activation and cytokine release. There are at least 26 staphylococcal SAg toxins distinguished, including the toxic shock syndrome toxin (TSST-1), enterotoxins and staphylococcal superantigen-like toxins (Oliveira et al., 2018; Vrieling et al., 2020).

The molecular pathogenesis of NF caused by *S. aureus* still remains poorly understood. Each exotoxin can cause different forms of tissue damage and inflammation. NF caused by *S. aureus* is relatively rare and as such there are few studies determining gene content based on complete genomes. Furthermore, knowledge of *S. aureus* virulence factor distribution in NF is insufficient. The aim of the present study was to identify virulence-related genes from a complete genome sequence of a methicillin-susceptible *S. aureus* isolate recovered from a monomicrobial case of NF. Moreover, we focused on the characterization of a new sequence variant of exfoliative toxin E, which can contribute to the severity and poor clinical outcome in NF patients.

## Materials and methods

### Case description

**FIGURE 1:**
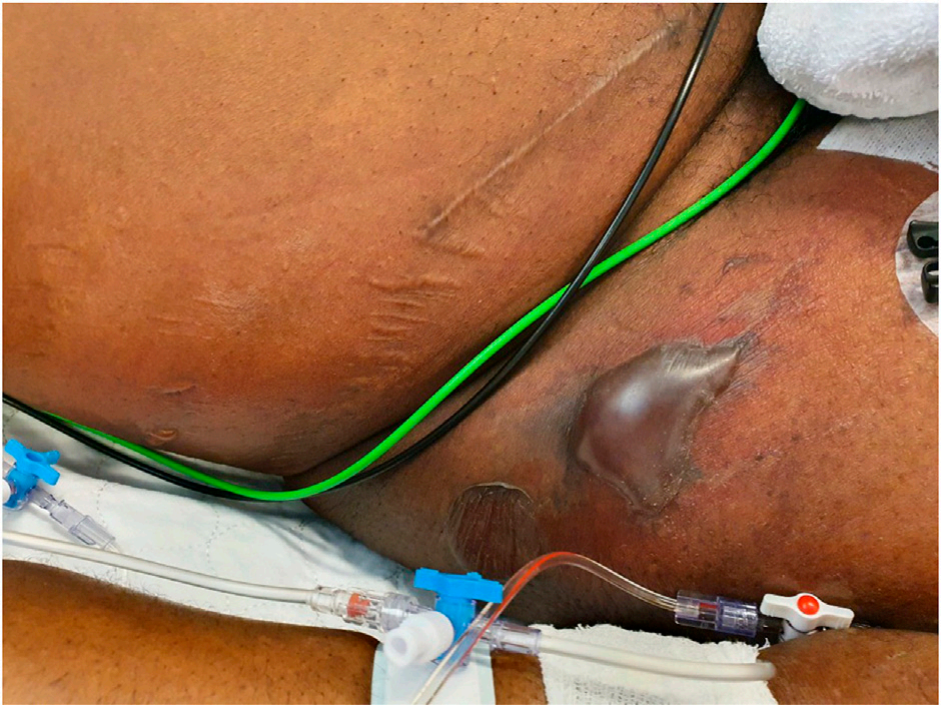
Clinical image of the right hip/inguinal region. Large superficial bullae appeared on purpuric lesions of the erythematous skin. Presumably, the bullae are caused by the systemic release of toxins by *S. aureus*, since no other causative pathogens were detected. A diagnosis of NF led to surgical debridement of necrotic tissue.

A 54-year-old male patient, born in Nigeria who had been living in the Netherlands for more than 10 years, presented at the emergency department of the University Hospital Center Groningen (UMCG) with septic shock: tachypnoea (30–40/min), tachycardia (120–130 beats/min) and hypotension (80/40). He had complaints of fatigue and fever starting 2 days before admission, and an abscess under the right axilla for a few days that had been incised and drained 1 day prior by his general practitioner. Physical examination revealed a purulent wound under the right axilla. The entire right flank felt indurated, pasty, and was painful on palpation. Doubtful minimal crepitus from this area of skin was elicited. Ultrasound investigation showed the appearance of cobblestoning, which can be indicative of cellulitis. The patient was immunocompromised: his medical history included HIV (2004), diabetes mellitus type II (2016) and kidney transplantation (2016). The patient was admitted to the intensive care ward. Antibiotic treatment was immediately started with cefuroxime 1500 mg three times daily, tobramycin 7 mg/kg once daily and clindamycin 600 mg three times daily, and mycofenolate mofetil was stopped. A pus sample from the axillary wound was cultured and grew *S. aureus*, which was resistant to penicillin and cotrimoxazole, and susceptible to all other antibiotics tested. Blood cultures grew *Brevibacterium spp* from one blood culture bottle, which was considered to be contamination. The next day the erythema had spread over the entire right flank to the lateral upper leg. On the third day of admission, several large and smaller tense bullae with a clear fluid content appeared on the erythematous skin of the lower flank to upper lateral leg. This area showed pitting edema and crepitus was elicited (Figure 1). No thermal or frictional damage had occurred in this area. The Nikolsky and pseudo-Nikolsky signs on the erythematous and healthy-looking skin were negative. All mucous membranes were normal. Fluid samples taken from the bullae tested negative for herpes simplex virus (HSV)-1, HSV-2, and varicella-zoster virus. HIV PCR on plasma showed no detectable load. Histology of a skin biopsy showed dermal edema and a diffuse infiltrate with neutrophils and no eosinophils. A skin biopsy for direct immunofluorescence was negative for auto-immune blistering diseases. A diagnosis of necrotizing fasciitis was made and surgical exploration and debridement of necrotic tissue was carried out. Samples from pus collections and deep tissue were cultured from which *S. aureus* grew in pure cultures. Blood cultures remained negative. Intravenous antibiotic treatment was switched to flucloxacillin 12 g/day *via* continuous infusion and vancomycin of which the dose was based on therapeutic drug monitoring. On day 3 and 5 after admission, surgical re-explorations were performed and further debridement of necrotic tissue from the right thoracic flank and abdomen was performed. On day 7, the patient was transferred to a burn center for skin grafting. Vancomycin was stopped, and treatment with flucloxacillin was continued. After prolonged treatment on the intensive care ward in the burn center, the patient recovered and was discharged to a nursing home. The patient provided his written informed consent to participate in this case study.

### Bacterial isolate

The *S. aureus* isolate UMCG579 was cultured from a pus collection from the subcutis of the patient on December 9th 2021. The isolate was initially cultured on Columbia agar with 5% sheep blood (BA incubated for 48 h at 35°C). Species determination was performed by Maldi-TOF (Brucker, Bremen, and Germany).

### Phenotypic antibiotic susceptibility testing

Automated susceptibility testing was performed by Vitek2 (bioMerieux, Marcy l'Etoile, and France). Susceptibilities were interpreted according to the current EUCAST breakpoints at the time of isolation. Susceptibility to penicillin was assessed by penicillin disk diffusion based on zone diameter and zone edge.

### Extraction of genomic DNA

Total DNA was isolated using the DNeasy Blood and Tissue Kit (Qiagen). DNA was quantified using Qubit 2.0 Fluorometer (ThermoFisher Scientific) and the quality assessed by TapeStation 2200 (Agilent Technologies). A NanoDrop 2000C spectrophotometer (ThermoFisher Scientific) was used to measure the purity of the extracted DNA.

### Whole genome sequencing

The genome of *S. aureus* isolate UMCG579 was sequenced with MinION Mk1C (Oxford Nanopore) and MiSeq (illumina) sequencers. The reads assembly was performed using SeqMan NGen version 18 (DNASTAR). Automated genome annotation was performed using the NCBI Prokaryotic Genome Annotation Pipeline (https://www.ncbi.nlm.nih.gov/genome/annotation_prok).

### Data analysis

The DNA sequences were aligned using BLASTn software (https://blast.ncbi.nlm.nih.gov/Blast.cgi). The MLST sequence type (ST) was assigned through the MLST server (Larsen et al., 2012). The *spa* type was determined with spaTyper (Bartels et al., 2014). The search for SCCmec elements was performed using SCCmecFinder 1.2 (Kaya et al., 2018). PHASTER (PHAge Search Tool Enhanced Release) was used to analyze prophages in the genome (Arndt et al., 2016). Genomic islands in the UMCG579 genome were identified using IslandViewer 4 (Bertelli et al., 2017). The amino acid sequence alignment and phylogenetic analysis of ETs were performed with the MegAlign Pro software (DNASTAR). SNP analysis was performed using the CSI Phylogeny 1.4 server (Kaas et al., 2014). The maximum likelihood tree produced by CSI Phylogeny 1.4 server was visualized in MEGA6 software (Tamura et al., 2013).

### Nucleotide sequence accession number

The sequence of the chromosome of *S. aureus* UMCG579 has been deposited in GenBank under accession number CP091066.

## Results

### Molecular characterization

The complete chromosome sequence of isolate UMCG579 consisted of 2,741,379 bp. UMCG579 did not harbor any plasmids. The chromosome showed a GC content of 32.86%, and contained 2527 coding sequences (with protein) and 83 RNAs. Based on *in silico* analysis, the isolate was identified as MLST ST1633, which belongs to clonal complex (CC) 152, and *spa* type t355. The search for SCC*mec* using the SCCmecFinder did not identify any SCC*mec* structure or its remnant DNA in this MSSA isolate. However, a sequence analysis of the UMCG579 chromosome using the IslandViewer webserver revealed five putative genomic islands (Supplementary Table S1). Among them there were two prophage regions. Analysis with the PHASTER webserver confirmed the existence of two intact prophages and additionally identified one incomplete prophage region in the genomic DNA of UMCG579 Supplementary Table S2).

### Toxin gene repertoire

To better understand pathogenic mechanisms of NF infections caused by *S. aureus,* we investigated the content of virulence genes of UMCG579 with a focus on searching for its unique genetic features harboring potent exotoxins. Virulence factor profiling identified multiple pore-forming toxin genes in the UMCG579 chromosome and none of the superantigen genes (Table 1). However, the *hla*, *hld* and *psmα* genes as well as the *hlg* gene cluster constitute the core genome and are shared by more than 95% of *S. aureus* strains (Grumann et al., 2014). Moreover, the *hlb*, *lukAB*/*GH* and *lukED* genes present in the UMCG579 chromosome are widespread and can be found in the majority of strains. The *lukF-PV* and *lukS-PV* genes, encoding subunits of Panton-Valentine leukocidin (PVL), are present in a small percentage (approximately 3%) of clinical *S. aureus* strains and they are strongly associated with community acquired MRSA strains, particularly those isolated from skin and soft tissue infections (Vandenesch et al., 2003). In the UMCG579 chromosome the PVL genes were located on an intact phage phi2958PVL (Supplementary Table S2).

**TABLE 1:** Major virulence genes in *S. aureus* UMCG579.

Virulence factor gene	Position in chromosome	Protein Function
Adherence		
*clfA*	773776..777057	clumping factor A
*clfB*	2640621..2643311 (complement)	clumping factor B
*Cna*	2718559..2722110 (complement)	collagen binding protein
*ebpS*	1436472..1437932 (complement)	elastin-binding protein EbpS
*fnbA*	2504569..2507826 (complement)	fibronectin-binding protein FnbA
*fnbB*	2501063..2503888 (complement)	fibronectin-binding protein FnbB
*sdrD*	532215..536324	serine-aspartate repeat protein D
Pore forming toxins		
*Hla*	1056230..1057189 (complement)	alpha-hemolysin precursor
*hlb*	2013235..2014227	beta-hemolysin
*hld*	2029162..2029242	delta-hemolysin
*hlgA*	2425223..2426152	gamma-hemolysin chain II precursor
*hlgB*	2427668..2428644	gamma-hemolysin component B precursor
*hlgC*	2426719..2427666	gamma-hemolysin component C
*lukD*	1882762..1883745 (complement)	leukocidin D component
*lukE*	1883746..1884681 (complement)	leukocidin E component
*lukA/lukG*	2014491..2015507 (complement)	leukocidin A/G component
*lukB/lukH*	2015529..2016557 (complement)	leukocidin B/H component
*lukF-PV*	1444463..1445440 (complement)	Panton-Valentine leukocidin F component
*lukS-PV*	1445442..1446380 (complement)	Panton-Valentine leukocidin S component
*psmα-1*	389955..390020 (complement)	phenol-soluble modulin PSM-alpha-1
*psmα-2*	389858..389923 (complement)	phenol-soluble modulin PSM-alpha-2
*psmα-3*	389738..389806 (complement)	phenol-soluble modulin PSM-alpha-3
*psmα-4*	389612..389674 (complement)	phenol-soluble modulin PSM-alpha-4
Exoenzymes		
*epiP*	1874291..1875664 (complement)	lantibiotic epidermin leader peptide processing serine protease EpiP
*sspA*	938970..939953 (complement)	Glu-specific serine endopeptidase SspA
*sspB*	937707..938888 (complement)	cysteine protease staphopain B
*scpA*	1984658..1985824	cysteine protease staphopain A
*aur*	2650383..2651912 (complement)	zinc metalloproteinase aureolysin
*ete2*	2223688..2224539 (complement)	exfoliative toxin E
*sak*	1829110..1829601 (complement)	Staphylokinase
*ednB*	2222714..2223469	epidermal cell differentiation inhibitor B
*hysA*	1819116..1821533	hyaluronate lyase
*lip1*	2700304..2702346 (complement)	YSIRK domain-containing triacylglycerol lipase Lip1
*lip2*	297349..299427	YSIRK domain-containing triacylglycerol lipase Lip2/Geh
*coa*	192697..194679	Staphylocoagulase
*nuc*	781276..781962	Thermonuclease family protein
Immune modulation		
*ebh*	1356399..1387838 (complement)	fibronectin binding protein ebh
*efb*	2423425..2424735	immunoglobulin-binding protein Sbi
*scn*	1827266..1827616 (complement)	complement inhibitor SCIN-A
*spa*		staphylococcal protein A
Biofilm		
*icaR*	2695830..2696390 (complement)	ica operon transcriptional regulator IcaR
*icaA*	2696554..2697792	poly-beta-1,6 N-acetyl-D-glucosamine synthase IcaA
*icaD*	2697756..2698061	intracellular adhesion protein IcaD
*icaB*	2698058..2698930	intercellular adhesin biosynthesis polysaccharide N-deacetylase
*icaC*	2698917..2699969	polysaccharide intercellular adhesin biosynthesis/export protein IcaC

### A new sequence variant of exfoliative toxin E

**FIGURE 2:**
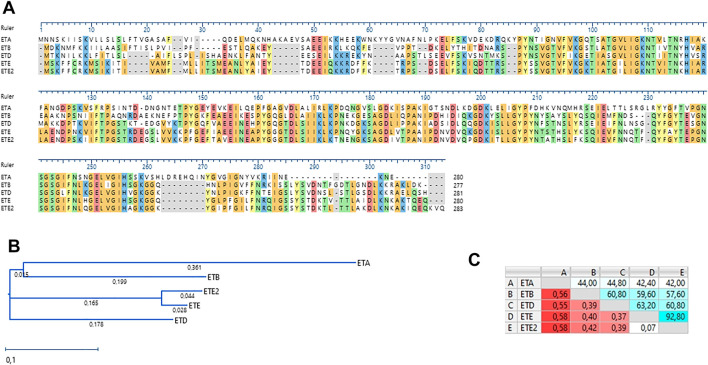
Amino acid sequence alignment and phylogenetic analysis of staphylococcal ETs recovered from human infections. **(A)** Amino acid sequence alignment (MUSCLE). **(B)** A neighbor-joining tree. The scale bar indicates the evolutionary distance between the sequences determined by 0.1 substitutions per amino acid at the variable positions. **(C)** Distance/%Identity among analyzed amino acid sequences.

Genome sequence analysis revealed that the UMCG579 isolate harbored a sequence variant of the recently described *ete* gene encoding a new exfoliative toxin (type E) (Imanishi et al., 2019). We propose to name this variant *ete2*. Comparison of the deduced amino acid sequences of the *ete* and *ete2* genes showed a 92.80% sequence identity (Figure 2A). Moreover, deduced sequence of the ETE2 protein was longer by three amino acids than that of ETE (283 AA versus 280 AA). The deduced amino acid sequences of the *ete* and *ete2* genes were compared to those of other ET proteins recovered from human infections (Figure 2B). The amino acid sequence of ETE showed higher sequence identity to ETA, ETB and ETD than that of ETE2 (Figure 2C).

### Prevalence of the *ete2* gene in *S. aureus* genomes

A BLAST search in the GenBank database revealed that the *ete2* gene was exclusively found among isolates (*n* = 115) belonging to MLST CC152; the majority of which were identified as ST152 (*n* = 112), with the remainder belonging to ST1633 (*n* = 2) and ST unknown (single locus variant of ST152, *n* = 1) (Supplementary Table S3). The isolates of CC152 possessing the *ete2* gene were recovered exclusively from human carriers or human infections. The deduced amino acid sequences of ETE2 were almost always (112 isolates) identical to that of the isolate UMCG579 or differed by only a single amino acid residue (3 isolates). We also investigated if we could identify *ete2*-negative isolates belonging to CC152. We found six ST152 isolates that did not harbor the *ete2* gene. They had a human origin and were isolated from nasal/nasopharynx swabs in Africa.

### Prevalence of the *ete* gene in *S. aureus* genomes

Analysis of the *ete* distribution using BLAST identified the presence of this gene mostly in MLST CC130 (*n* = 123) and also in several genetically unrelated STs (*n* = 10), including ST890, ST2233, ST2867, ST2970, and ST2990 (Supplementary Table S4). Unlike *ete2*, the *ete* gene was found not only in human sources (*n* = 55) but also in animals (*n* = 71) including; European red deer (*Cervus elaphus)* (*n* = 18), domestic pig (*Sus scrofa domesticus*) (*n* = 16), domestic sheep (*Ovis aries*) (*n* = 12) European red squirrel (*Sciurus vulgaris*) (*n* = 11), domestic goat (*Capra hircus)* (*n* = 8), domestic cow (*Bos taurus*) (*n* = 3), wild goat (*Capra hircus*) (*n* = 2) and European hedgehog (*Erinaceus europaeus*) (*n* = 1). Like *ete2*, the *ete* gene was also highly conserved independently of genetic background (different MLST CCs) or animal source of isolation.

The deduced amino acid sequences of ETE were identical to that of the isolate O46 or differed by up to three amino acid residues.

### Genetic organization of genomic island harboring the *ete2* gene

**FIGURE 3:**
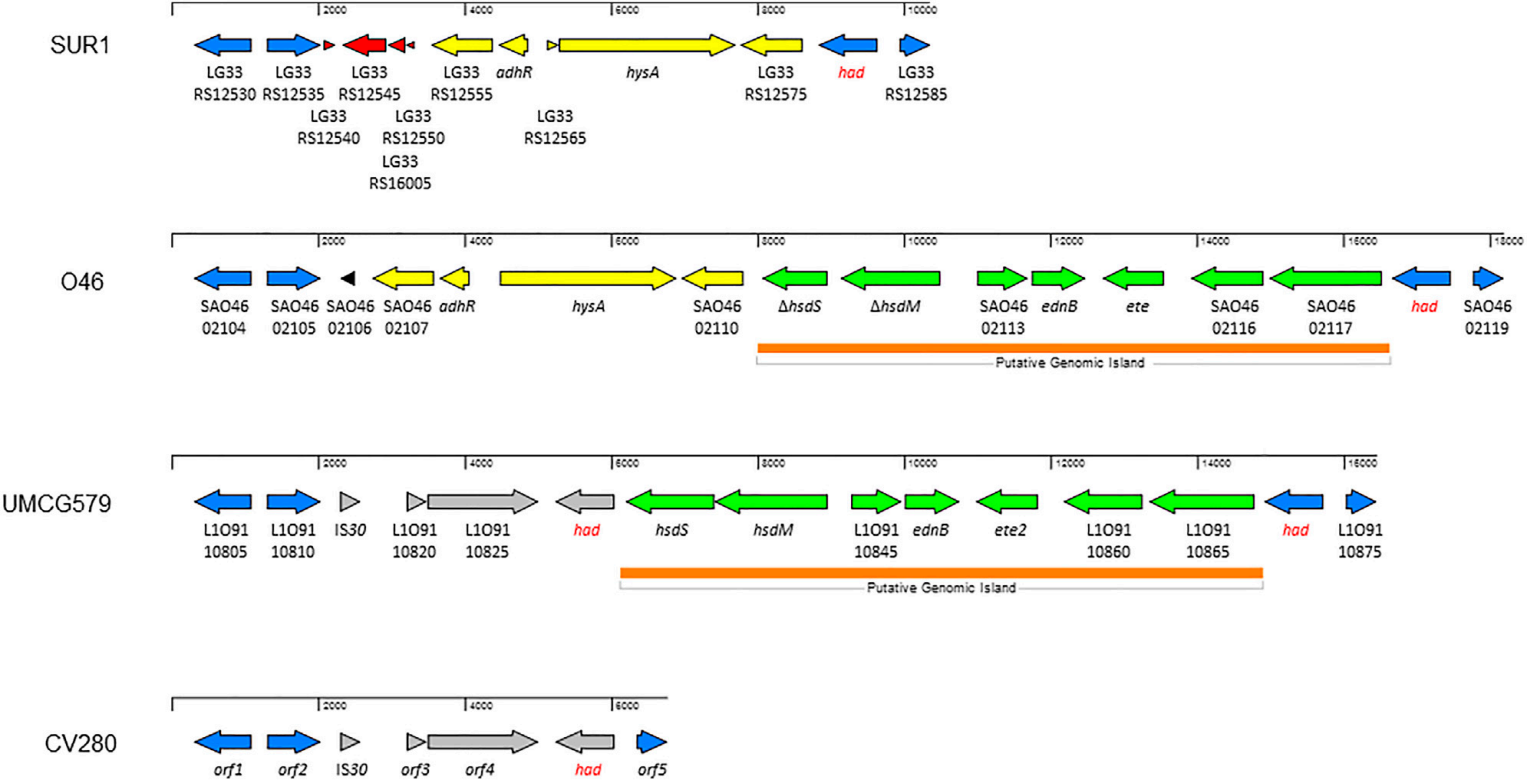
Structure of the genomic islands containing the *ete* and *ete2* genes from *S. aureus* isolates O46 (GenBank accession number CP025395) and UMCG579 (GenBank accession number CP091066), respectively; and comparison with genome sequences of isolates USA300_SUR1 (GenBank accession number CP009423) and CV280 (GenBank accession number JAAWZQ000000000). The arrows indicate open reading frames and their orientations. Blue arrows: genes shared by isolates SUR1, O46, and UMCG579. Yellow arrows: genes shared by isolates SUR1 and O46. Green arrows: genes shared by isolates O46 and UMCG579. Grey arrows: genes shared by isolates O46 and CV280. Red and black arrows: genes only present in isolate SUR1 or O46, respectively. ORFs are described further in Table 2.

To determine the size and gene content of a putative genomic island harboring the *ete2* gene, the genome sequence of UMCG579 was compared to that of the O46 isolate (GenBank accession number CP025395) containing a genomic island with the *ete* gene (Figure 3). Moreover, to determine accurate boundaries of the island, the UMCG579 genome sequence was compared to that of a genetically unrelated (MLST ST8) and exfoliative toxin negative SUR1 isolate (GenBank accession number CP009423), representative of the USA300 lineage. We searched in both UMCG579 and O46 isolates a string of genes in the vicinity of an exfoliative toxin gene that were not present in the SUR1 isolate. Comparative genome analysis revealed that the *ete2* gene was located on a 8777 bp putative genomic island (Figure 3). This island contained six other genes encoding proteins associated with a restriction-modification system (*hsdS* and *hsdM*), virulence (*ednB*), proteolysis (L1O91_10845) and cellular activities (L1O91_10860 and L1O91_10865) (Table 2). Nucleotide alignment of the island containing the *ete2* gene of UMCG579 revealed that it shared 95.67% nucleotide identity with a genomic island of the O46 isolate over 95% of its length. The most likely insertion site of the *ete2*-containing island in the UMCG579 chromosome was the *had* gene encoding HAD-IIB family hydrolase as the island was bound by two copies of this gene in direct orientation. This configuration could have arisen if the *had* gene in a circular molecule recombined with the *had* gene already present in the chromosome through homologous recombination. Two copies of the *had* gene (816 nucleotides in length) were not identical differing by nine nucleotides. Moreover, the copy of the *had* gene located upstream of *ete2* possessed a mutation causing a premature stop codon, while the *had* copy located downstream was fully functional. Homologous recombination also generated a deletion variant, which was found in six CC152 isolates. The deletion arose through recombination between the two copies of the duplicated *had* gene resulting in a loss of all genes of the *ete2* genomic island (Figure 3).

**TABLE 2:** Genes present in the genomic islands containing *ete* and *ete2* genes.

Isolate SUR1 gene name	Isolate O46 gene name	Isolate UMCG579 gene name	Isolate CV280 gene name	Product
LG33_RS12530	SAO46_02104	L1O91_10805	*orf1*	DeoR/GlpR family DNA-binding transcription regulator
LG33_RS12535	SAO46_02105	L1O91_10810	*orf2*	NAD-dependent protein deacylase
LG33_RS12540	Absent	Absent	Absent	Hypothetical protein
LG33_RS12545 (frameshifted)	Absent	Absent	Absent	DUF3885 domain-containing protein
LG33_RS16005 (internal stop)	Absent	Absent	Absent	SAR2788 family putative toxin
LG33_RS12550	Absent	Absent	Absent	Hypothetical protein
Absent	SAO46_02106	Absent	Absent	Hypothetical protein
LG33_RS12555	SAO46_02107	Absent	Absent	Aldo/keto reductase
*adhR*	*adhR*	Absent	Absent	Transcriptional regulator, MerR family
LG33_RS12565	Sequence present but not annotated	Absent	Absent	Hypothetical protein
*hysA*	*hysA*	Absent	Absent	Hyaluronate lyase
LG33_RS12575	SAO46_02110	Absent	Absent	M23/M37 peptidase domain protein
Absent	Absent	IS*30* (frameshifted)	IS*30* (frameshifted)	IS30 family transposase
Absent	Absent	L1O91_10820	*orf3*	Hypothetical protein
Absent	Absent	L1O91_10825	*orf4*	Glycosyltransferase
Absent	Absent	*had* (internal stop)	*had* (internal stop)	HAD-IIB family hydrolase
Absent	*hsdS* (frameshifted)	*hsdS*	Absent	Type I restriction-modification system, restriction endonuclease subunit S
Absent	*hsdM* (partial deletion)	*hsdM*	Absent	Type I restriction-modification system subunit M
Absent	SAO46_02113	L1O91_10845	Absent	Trypsin-like serine protease
Absent	*ednB*	*ednB*	Absent	Epidermal cell differentiation inhibitor
Absent	*ete*	*ete*2	Absent	Exfoliative toxin type E
Absent	SAO46_02116	L1O91_10860	Absent	AAA family atpase
Absent	SAO46_02117	L1O91_10865	Absent	AAA family atpase
*had*	*had*	*had*	Absent	HAD-IIB family hydrolase
LG33_RS12585	SAO46_02119	L1O91_10875	*orf5*	MAP domain-containing protein

### Single nucleotide polymorphism analysis

**FIGURE 4:**
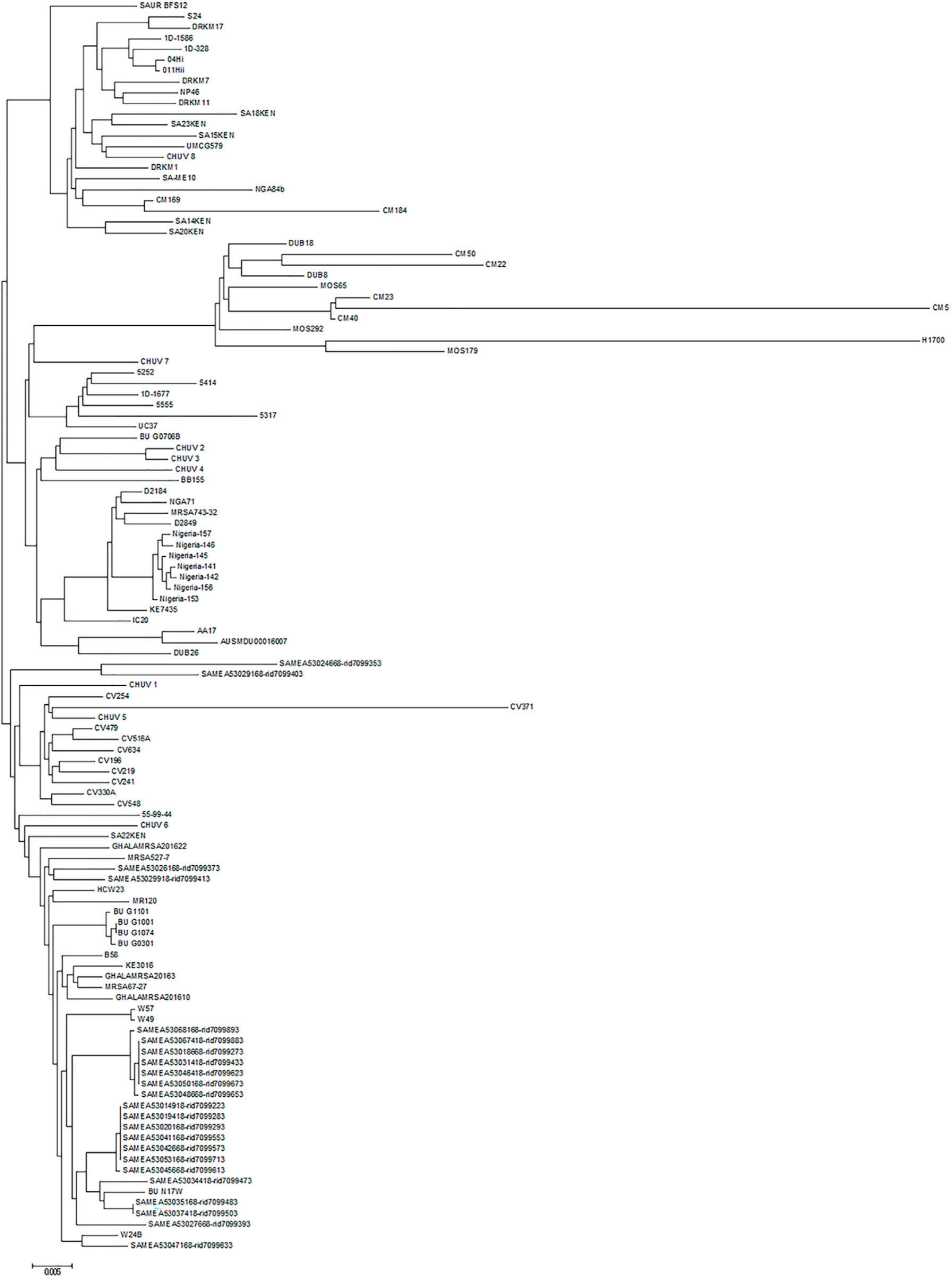
Phylogenetic relationship of *S. aureus* CC152 isolates containing the *ete2* gene. Maximum likelihood tree was constructed on the basis of genome-wide single nucleotide polymorphism (SNP) analysis. The scale bar indicates the evolutionary distance between the sequences determined by 0.005 substitutions per nucleotide at the variable positions.

The chromosome sequences of all *ete2*-positive isolates (*n* = 116) were uploaded to the CSI Phylogeny 1.4 server (https://cge.cbs.dtu.dk/services/CSIPhylogeny) in order to investigate their single nucleotide polymorphism (SNP)-based phylogeny. Genome-wide SNP results revealed that the UMCG579 chromosome sequence was most closely related to that of CHUV 8 and SA15KEN (Figure 4), although the sequences differed by 82 and 106 SNPs, respectively. Isolates CHUV 8 and SA15KEN were recovered 6 years earlier than UMCG579, and they had different geographic origins, from Switzerland and Kenya, respectively.

## Discussion

For adequate and cost-effective infection prevention, it is important to identify *S. aureus* strains based on their virulence gene profiles that may be more aggressive and have a higher potential to establish a life-threatening infection. Two potent toxins that are heterogeneously distributed among *S. aureus* strains were identified in the UMCG579 genome, PVL and ETE2. PVL is a cytotoxin that lyses human polymorphonuclear leukocytes, especially neutrophils, and intradermal injection of purified PVL to rabbits causes edema, erythema and tissue necrosis (Cribier et al., 1992; Löffler et al., 2010). Moreover, epidemiological data demonstrates compelling evidence for PVL as an important virulence factor of *S. aureus* in severe skin and soft tissue infections (SSTI) and necrotizing pneumonia in humans (Gillet et al., 2007; Gillet et al., 2008; Nurjadi et al., 2015). However, several experimental studies in animal models of SSTI contradict each other. Despite evidence from a study that showed using a PVL-positive and its isogenic PVL-knockout strain that PVL plays a role in tissue pathology in the early stages of infection (Lipinska et al., 2011), other studies could not detect a contribution of this toxin in necrotizing skin infections (Li et al., 2009; Kobayashi et al., 2011).

The production of ETs in *S. aureus* strains is associated with superficial infections such as localized bullous impetigo and its generalized form staphylococcal scalded skin syndrome (SSSS) (Oliveira et al., 2018). All *S. aureus* ETs are unique glutamate-specific serine proteases that specifically cleave a peptide bond after glutamic acid residue 381, which is located in the extracellular segment of human and murine desmoglein 1 (Hanakawa et al., 2004; Bukowski et al., 2010; Imanishi et al., 2019). This cleavage causes efficient and specific abolishment of keratinocyte junctions and cell-cell adhesion in the epidermis resulting in host skin damage (Nishifuji et al., 2008). Necrotizing infections can occur after minor or major breaches of the skin (Stevens et al., 2021). Furthermore, deep-seated infections are frequently associated with *S. aureus* strains that produce PVL (Holmes et al., 2005). Therefore, the combined production of ETE2 and PVL may increase the risk of the occurrence of more severe symptoms during infection and be associated with poor clinical outcome in NF patients.

Conflicting results obtained in different studies regarding the role of PVL in necrotizing skin infections may result from the presence of different virulence factor profiles in the genomic DNA of *S. aureus* strains. *S. aureus* produces numerous and often functionally redundant virulence factors. Therefore, it is important to examine the full gene content from the complete genome sequences to better understand the contribution of particular *S. aureus* virulence determinants to NF infection. However, only a limited number of *S. aureus* isolates recovered from NF patients have been investigated by whole-genome sequencing (WGS). Takano and others reported that ST764 MRSA, which is a local variant of the ST5 lineage, caused invasive infections, including necrotizing fasciitis (Takano et al., 2013). Analysis of the genome sequence revealed that ST764 carried the staphylococcal enterotoxin genes *seb*, *seg*, *sei*, *sem*, *sen*, and *seo*, and was negative for the PVL genes. In another study, Bakthavatchalam and others showed genomic insights into MRSA strain of ST772 (belonging to CC1) and *spa* type t657 from a case of fatal necrotizing fasciitis (Bakthavatchalam et al., 2018). Analysis of the genome for virulence determinants revealed the presence of staphylococcal enterotoxin genes *sea*, *seg*, *sei*, *sel*, *sem*, *seo*, and *sec3*. Moreover, the genome of ST772/t657 MRSA strain possessed the prophage phiSa119 carrying an operon with the *lukS*/*F*-PV genes encoding PVL. Aswani and others analyzed the genome sequence of ST45 MSSA strain that caused severe necrotizing fasciitis in a 72-year-old diabetic male (Aswani et al., 2019). The toxin landscape of the ST45 MSSA strain consisted of the enterotoxin gene cluster (*egc*): *seg*, *sen*, *seu*, *sei*, *sem*, and *seo*, as well as the *eta* gene encoding exfoliative toxin A. The strain was lacking the genes for PVL. Furthermore, Yamagishi et al. (2020) and others presented a case of monomicrobial Fournier’s gangrene (NF of the perineal, perianal or genital regions) caused by MSSA belonging to ST8 and *spa* type t622. WGS and a search for virulence factors revealed that the ST8/t622 strain harbored the *tst* gene encoding toxic shock syndrome toxin-1 (TSST-1) and was PVL-negative.

The PVL-positive ST152 lineage seems to be a hyperepidemic community associated MSSA in Africa and associated with chronic wound infections (Wolters et al., 2020). This lineage is especially prevalent in west and central Africa (Schaumburg et al., 2014; Obasuyi et al., 2020). Recent study by Obasuyi et al. (2020) and others showed that ST152-MSSA was the most dominant MLST ST (accounting for 24.7% of all MSSA isolates) in Benin City in South-South Nigeria. This finding was in line with another study by Okon et al. (2014) and others, who found that the predominant MSSA strain recovered in tertiary-care hospitals in North-East Nigeria was ST152-MSSA. CC152 strains are not found widely outside of Africa and have been only occasionally isolated from patients in some European countries.

In this paper we showed that the UMCG579 genome contained a novel sequence variant of exfoliative toxin E (ETE2) located on a genomic island, which was acquired through homologous recombination. The amino acid sequence of ETE2 differed by 7.2% from that of ETE being also longer by 3 amino acid residues. It warrants further study to compare the biochemical properties of the two variants of this exfoliative toxin such as host-specific activity, pH optimum, thermostability or inhibition studies of the enzymes by natural and synthetic inhibitors. While the majority of *S. aureus ete*-positive isolates were recovered from animal sources, *S. aureus ete2*-positive isolates had a human origin. Most infections caused by *S. aureus* are due to the combined action of various factors. The combination of two potent toxins, ETE2 and PVL, is likely to enhance the virulence of *S. aureus* isolates.

## Data Availability

The datasets for this article are not publicly available due to concerns regarding participant/patient anonymity. Requests to access the datasets should be directed to the corresponding author.
